# Impact of a Nutrition-Related Community Intervention on the Quantity and Quality of Children’s School *almuerzo*

**DOI:** 10.3390/life11030253

**Published:** 2021-03-19

**Authors:** Jenny Vilchis-Gil, Miguel Klünder-Klünder, Ximena Duque, Gloria Martínez-Andrade, Andrea Martínez-Almaráz, Brenda Beristain-Lujano, Samuel Flores-Huerta

**Affiliations:** 1 Epidemiological Research Unit in Endocrinology and Nutrition, Hospital Infantil de México Federico Gomez, Ministry of Health (SSA), 06720 Mexico City, Mexico; andy_270293@hotmail.com (A.M.-A.); brendaberistain10@gmail.com (B.B.-L.); sflores@himfg.edu.mx (S.F.-H.); 2 Medicine Faculty, National Autonomous University of Mexico, 06320 Mexico City, Mexico; 3 Deputy Director of Research, Mexico Children’s Hospital Federico Gómez, 06720 Mexico City, Mexico; miguel.klunder@himfg.edu.mx; 4 Research Committee, Latin American Society for Pediatric Gastroenterology, Hepatology and Nutrition (LASPGHAN), Mexico City, Mexico; 5 Infectious Diseases Research Unit, Pediatric Hospital, Mexican Institute of Social Security, 06600 Mexico City, Mexico; ximena.duque@imss.gob.mx; 6 Epidemiological and Health Services Research Unit, Mexican Institute of Social Security, 06600 Mexico City, Mexico; gloria.martineza@imss.gob.mx

**Keywords:** child, school *almuerzo*, early intervention, prevention, obesity

## Abstract

Foods and beverages that schoolchildren carry in their lunchboxes have high energy values but lack plain water, fresh fruits and vegetables. A nutrition-related community intervention on the quantity and quality of school *almuerzo* was performed, in which four primary schools participated, as part of two groups: 225 children in the intervention group (IG) and 177 children in the control group (CG). The parents from the IG had access to a website where they could consult information on eating habits and physical activity or school *almuerzo* menus. They were sent weekly text messages on their mobile phones and attended in-person sessions. Anthropometric measurements and surveys were performed in both groups at the start of the study, as well as after 6 and 12 months. The school *almuerzo* was assessed by recording foods that the children brought in their lunchboxes. At baseline, 88% of children brought a school *almuerzo*, 37% fruit, 17% vegetables, 40% plain water and 50% sweet drinks. In both groups, 50% of children brought a school *almuerzo* with an energy value above the recommended value (>340 kcal) during follow-up; however, the percentage of children who brought sweet drinks decreased (*p* < 0.05), with sweet drinks contributing between 26% and 33% of the calories in the school *almuerzo*. In the IG, the quantity in milliliters of plain water increased at the end of the follow-up period (*p* = 0.044). From the point of view of food-and-beverage quantity and quality, school *almuerzo* were unhealthy for both groups. The intervention failed to increase the frequency with which parents provided children with school *almuerzo* or enhance the quality of the latter.

## 1. Introduction

Childhood obesity is a public-health issue that has recently increased, thus continuing to represent a health challenge owing to comorbidities developed between childhood and adulthood. Obesity is the excessive accumulation of adipose tissue, primarily attributed to an energy disequilibrium characterized by a positive balance of energy that occurs when the energy intake exceeds the energy expenditure, leading to an increase in body-fat deposits, weight gain, and the risk of developing metabolic and inflammatory diseases from an early age [1].

According to the 2018 National Survey for Health and Nutrition in Mexico, the prevalence of overweight and obesity in children aged between 5 and 11 was 35.5%; although it has not augmented within the past 10 years, it continues to be unacceptably high [2]. When it comes to obesity—multifactorial inflammatory state—eating habits and physical activity play a predominant role, particularly among schoolchildren [3]. In Mexico, the government educational system does not provide children with food during the time they spend at school; therefore, their nourishment depends on the refreshments provided by their parents or on the food that they buy at the school store and eat during their 30 minutes’ break.

The school “*almuerzo*” is an eating occasion considered one of the main meals of the day. In Mexico, the main meals for school children are (in Spanish): *desayuno, almuerzo, comida y cena* (breakfast, brunch, lunch and dinner). *Almuerzo* is one main meal that the children have at the break in school [4,5]. In Mexico, the children are at school from 7:30–8:00 am until 12:30–13:00 h, and they have their breakfast and “*comida*” at home at 6:30 and 15:00 h, on average. Mexico’s Guide for Nourishment in Public Primary Schools states that, for a school meal to be healthy, it must account for between 15% and 20% of the daily energy recommendation and include one serving of fruits and/or vegetables, one serving of cereal or tubers, one serving of leguminous plants and/or animal-source food, and plain water [6]. In Mexico, the school *almuerzo* accounts on an average for 24% of the daily energy recommendation for pupils, regardless of their age, sex and physical activity [6].

The prevention of overweight and obesity is a necessary alternative, which in the long term becomes less expensive than the impact of the sicknesses caused by them. Note that both in developed and developing countries, school meals are composed of densely energetic foods with a high content of saturated fat, sucrose and fructose, which is common in processed foods, but with a low nutritional value, as well as low contents of fruits, vegetables and plain water [6,7].

Primary schools are environments that offer the opportunity to put together formative interventions aiming to achieve the prevention of overweight and obesity through actions that involve both the children and their parents in the process of making decisions related to selecting, buying, preparing and consuming food, as well as acquiring healthy eating habits, especially when it comes to enhancing the quantity and quality of the foods and beverages that comprise the school *almuerzo*. Our study aimed to assess the impact of such a formative intervention on the quantity and quality of foods and beverages that comprise the school *almuerzo* over the course of a year.

## 2. Materials and Methods

### 2.1. Design and Study Population

This study is part of a formative intervention aimed at parents of schoolchildren, both in-person and remotely, which deals with proper eating habits and physical activity. The primary aim of the intervention was to assess the change in the children’s body mass index (BMI z-score) [8]. This study highlights the results of the analysis of the quantity and quality of foods and beverages consumed within the intervention group (IG) and the control group (CG), as well as the differences noticed between the baseline assessment and the follow-up period of 6 and 12 months. Pupils and parents from four primary schools with approximately the same number of pupils—two public and two private—in Mexico City participated in this study. The protocol of this study was approved by the committees for the Investigation, Ethics and Biosecurity from Mexico’s Federico Gómez Hospital for Children (HIM/2013/003); the study was authorized by Mexico’s Ministry of Public Education and the principals of the schools that participated in this study. Written informed consent was provided both by parents and children. The study comprised boys and girls between first and fourth grade, enrolled at the selected primary schools. We excluded children that were participating in weight-loss programs or suffered from chronic diseases. Children with low weight and without school *almuerzo* records were excluded from the analysis.

### 2.2. Implementing the Intervention

We presented the aims and activities of our “Aliméntate y Actívate Sanamente” (“Nourish and Activate Yourself Healthily”) study to the children, their parents and their teachers at the primary schools that were selected [8]. We implemented our formative intervention in-person and remotely during the 2013–2014 school year, with the purpose of improving diet habits and conducts—selecting, buying and preparing food and food servings—inside and outside the home, thus increasing physical activity and reducing the sedentary lifestyles of individuals and families.

#### 2.2.1. Intervention Schools

In-person and remote activities with the children’s parents. During the intervention, the children’s parents had access to a website where they could identify: (a) Topics related to nourishment and physical activities, which were changed every 15 days (there were 20 topics in total). During each topic, the parents were asked to perform certain activities inside or outside their homes with the purpose of maintaining or enhancing their eating habits and their physical activity (Table S1) (b) School *almuerzo*. An information manual on how to provide a healthy school meal with examples; (c) Two guides on various healthy-nourishment and physical-activity topics, which were provided to the parents both in print and digitally on the website; (d) Nine posters, which were posted in the schools with the purpose of providing information on nourishment and physical activity to the children—information that could be found on the website; (e) Software for calculating the BMI. On the website, both the parents and their children could assess their health state throughout the intervention; (f) Means of contacting the person responsible for the project, in case the parents had doubts or comments about how easy or difficult it was for them to perform the activities.

The parents received weekly text messages on their mobile phones related to nourishment and physical activity, to motivate and strengthen the fostered habits and conducts. Throughout the intervention, 40 text messages were sent (Table S1).

In-person activities with the parents. The parents were invited to three educational sessions of 40–60 min—one session every two months—that were lectured by two nutritionists and a certified physical-education teacher, and took place at different times in each school. The first session focused on the risk of obesity and the Mexican Eat Well Plate, the second on the importance of breakfast and school *almuerzo*, and the third on consuming fiber and plain water. During each of the three sessions, 20 min were dedicated to doing physical activity with the parents. At the end, the parents received two printed guides on healthy nourishment and physical activity.

In-person activities with the children. The nutritionists organized four 50 min workshops—one every two months—over the school year, during which they tackled topics related to nourishment and physical activity. Each child was presented with two laminated tablecloths: one displaying the Mexican Eat Well Plate, whereas the other was displaying the Physical Activity Pyramid. The former represents Mexico’s nourishment guide for health promotion and education, which establishes criteria related to nutritional orientation and highlights each of the food groups, to present the variety of foods and the proper way to combine them for a balanced diet [9]. Moreover, the children and their parents visited the Health Hall at the UNIVERSUM—the UNAM’s (National Autonomous University of Mexico) Museum of Sciences—which displays activities related to proper nourishment.

Activities at school. A poster was posted every month in strategic locations around the schools, such as the entrances, the patios and the halls, with the purpose of fostering healthy diets, water-drinking and exercise, both at school and at home.

#### 2.2.2. Control Schools

In the CG schools, the same anthropometric measurements and surveys were performed as in the IG schools. After measurements, the parents received a record of these, along with a nutritional diagnosis and a sheet of paper containing fundamental recommendations meant to help them enhance their eating habits and methods of performing physical exercise.

### 2.3. Measurements

#### 2.3.1. Sociodemographic Data

We obtained this information using a questionnaire, which the children delivered to their parents and which the parents subsequently returned later the same week. The questionnaire comprised questions related to what goods the family possessed such as TVs, refrigerators, washing machines, ovens, heaters, landlines, computers, internet or cars. The homes were split into three categories considering the score they obtained at a socioeconomic level after analyzing their primary components. According to the economic level of the households, these categories were low, average and high. Furthermore, we obtained information regarding the mothers’ level of education considering the degree reached or completed. We categorized this information into secondary or less education, high school or technical school and college career or postgraduate.

#### 2.3.2. Anthropometric Measurements

These measurements were performed by two nutritionists who participated in a certification and standardization process. Regarding the baseline period of 6 and 12 months, the weight and body height of the children in the IG and CG were measured. Briefly, the children were measured without shoes and in light clothing. They stood, with their weight equally distributed on both feet, and their arms resting freely at their sides. The children’s body height was measured using a Seca 225 stadiometer (Seca Corp., Hamburg, Germany), and recorded in centimeters with an accuracy of 0.1 cm in accordance with standard international procedures [10]. The children’s weight was measured using a Seca 882 digital scale (Seca Corp., Hamburg, Germany) with an accuracy of 0.1 kg with the children required to stand in the center of the platform while the measurement was performed.

The BMI *z-score* was calculated in accordance with the 2007 reference data provided by the World Health Organization [11]. The children were sorted as follows: weight normal (−2 < *z-score* < 1), overweight (1 ≤ *z-score* < 2) and obesity (*z-score* ≥ 2).

#### 2.3.3. Questionnaire Regarding Eating Habits and School *almuerzo*

A questionnaire was applied by two certified nutritionists to the children comprising both groups at the beginning of the study and subsequently after 6 and 12 months. On the day of the anthropometric measurements, the children were interviewed during their school break in a place established by the principals of each schools. Information was obtained regarding whether the children had brought their own meals and/or money to buy food at school. Then, the children were sorted into those who had brought their own meals at school, those who had brought both meals and money to buy extra food at school, those who had only brought money and those who had not brought anything.

Similarly, a record was maintained of the foods and beverages present in the children’s lunchboxes without considering the food bought by the children before arriving at school. The children were required to open their lunchboxes and they were given the reason why information was required on what foods they were carrying within. After the children opened their lunchboxes, the two certified nutritionists wrote down what foods and beverages they found inside, describing the ingredients in each of them, as well as the estimated quantity of these. The foods and beverages were recorded in grams or milliliters, servings (piece, plate, spoonful, teaspoonful, cup, and slices), number of servings, characteristics of the food, beverage or preparation (brand, cooking type). For industrial products, the two nutritionists recorded the grams and milliliters displayed on the product.

The quantity of the school *almuerzo* was measured using its energy value and content of macronutrients. The software Food Processor SQL (version 10.9.0, 2011, ESHA Research, Salem, OR, USA) was used to determine the energy value, carbohydrates, lipids and proteins present in the school meals of the children who took part in the three measurements. The school meal’s energy value in kilocalories (kcal) was calculated and categorized according to the guidelines provided in the Technical Document of Recommendations for Nourishment Guides in Public Primary Schools [6]; departing from the energy value, we devised four categories: (a) no school meal, (b) low in kcal (<250 kcal), (c) healthy (between 250 and 340 kcal, equal to an average energy value of between 15% and 20% of the daily recommended one, defined as a healthy school meal within this population) and (d) high in kcal (>340 kcal). We also compared the energy value of the school meals brought by some of the children as their only meal to that of school meals brought by other children in addition to money for extra food.

To assess the quality of foods and beverages present in school *almuerzo*, these were sorted according to the similarities between the macronutrient contents within. We obtained the following categories: (a) fruits (e.g., apple, banana, grapes, guava, kiwi, melon, orange, papaya, peach, pear, pineapple, plums, tangerines, strawberries, and watermelon); (b) vegetables (e.g., broccoli, prickly pear, carrot, corn pimples, cucumber, yam beans, lettuce, onion, chayote, tomato, alfalfa germ, canned vegetables, peas, beet, hot pepper, cauliflower, and celery); (c) sweet drinks (fruit water with sugar, industrialized juice, natural juice, refreshing drinks, flavored water, and powder-source water), not including sugar-free beverages; (d) cookies and cereal boxes (chocolate-chip cookies, sprinkle sugar cookies, sandwich-style cookies, chocolate cereal bars, apple, strawberry and oats, salty cookies, cookies with filling, with or without chocolate glazing, chocolate cake, chocolate bars, cereal box, donuts, and animal-shaped biscuits); (e) dairy products (whole milk, flavored or not, yogurt, fresh cheeses-panela cheese, cottage cheese, cream cheese—or matured cheeses—or American); and (f) plain water. To assess the quality of school meal, we considered assessing the percentage of children who brought fruits, vegetables, sweet drinks, cookies and cereal boxes, dairy products and plain water. Moreover, we determined the quantity in grams and/or milliliters, in addition to the caloric content of each of the food groups, thus generating the caloric-percentage variable that each of them contributed to the overall energy value of school meal.

### 2.4. Statistical Analysis

Descriptive statistics was used to compare the characteristics of the study population, as well as the energy value and macronutrients within the school meals, to the baseline period. The children’s mean weight and body height were adjusted as per age and sex, in a multiple linear regression. To compare the study groups to the baseline period, for continuous variables we employed Student’s *t*-test for independent data and, for categorical data, we used Pearson’s Chi-squared test. To compare the energy value and the grams and percentage of macronutrients (carbohydrates, lipids and proteins) present in the groups’ school meals to the baseline period, the Mann–Whitney U test was used.

To assess the intra-group change in the habits related to bringing a school meal along with money to buy food at school, as well as the frequency of foods and beverages comprising a school meal and of the energy-value categories within each group, the test of equality of proportions for paired samples (Cochran’s Q) was used.

The ANOVA model was used for repeated measures and marginal prediction to perform an intra-group comparison between the average food-group consumption in grams and milliliters, and the percentage of calories contained within each food group comprising the school meal. We assigned *p* < 0.05 values as statistically significant for all the analyses. The analysis was performed using Stata v12.0 (Stata Corp., College Station, TX, USA).

## 3. Results

In this study, 407 children agreed to participate, of which 226 comprised the IG and 181 the CG. One child from the IG and four children from the CG were excluded from the analysis because of a lack of information regarding their school *almuerzo*; therefore, 225 children in the IG and 177 children in the CG were included in the analysis. At the end of the study, there were 86.6% children left in the IG and 84.1% in the CG. The losses incurred during the follow-up period were primarily attributed to the children’s switching schools. In the IG, 51.5% of the parents (116 out of 225) attended at least one educational session at the school, 40.9% (92 out of 225) surfed the website, and 91.5% (206 out of 225) received the text messages on their mobile phones. All of the children attended the workshops [8].

At the start of the study, the anthropometric and demographic characteristics were similar in both groups. The mean schoolchildren age was 8.0 ± 1.2 years old and the children’s BMI z-score was 0.85 ± 1.4 in the IG and 0.99 ± 1.3 in the CG, 19.6% and 25.4% of the children presented obesity in the IG and CG, respectively, without statistically significant differences. Moreover, 41% of mothers reported having education levels equal to a bachelor’s or master’s degree (Table 1).

**Figure 1:**
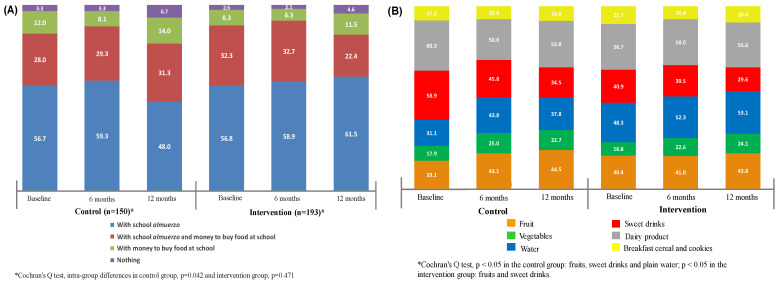
(**A**) Change in the percentage of children who brought school *almuerzo* and money to buy food at school during follow-up. (**B**) Frequency of foods and beverages comprising the school *almuerzo*, during the follow-up period.

In the IG, 89.1%, 91.6% and 83.4% of children brought their own school meal, while 32.3%, 32.7% and 22.4% brought money to buy extra food during the baseline period of 6 and 12 months. In the CG, 84.7%, 88.6% and 79.3% of the children, during the baseline period of 6 and 12 months, brought their own school meal, with 28%, 29.3% and 31.3% having brought money to buy extra food at school (Figure 1A). When assessing the quality of foods comprising the school meals during the follow-up year (Figure 1B), we noticed that a low percentage of children from both groups brought fruits, vegetables and plain water, while a high percentage of children brought sweet drinks. In the CG, the percentage of children who brought fruits slightly increased; however, in the IG, the percentage remained the same during the follow-up period (CG: 33.1%, 43.1% and 44.5%; IG: 40.4%, 41% and 43.8% at baseline, 6 and 12 months, respectively). Furthermore, the percentage of sweet drinks decreased in both groups (CG: 58.9%, 45.8% and 34.5%; IG: 40.9%, 39.5% and 29.6% at baseline, 6 and 12 months, respectively); however, the percentage of plain water remained steady (CG: 31.1%, 43.8% and 37.8%; IG: 48.3%, 52.3% and 53.1% at baseline, 6 and 12 months, respectively). For both groups, <25% of the children brought vegetables in their school meals during the follow-up period (Figure 1B).

At the start of the study, any differences related to the energy value and the macronutrients were found comprising the school *almuerzo* between the CG and the IG, with the average energy value higher than the one recommended for a healthy school meal [6], namely, 395 kcal; IQR:281-527 kcal (Table 2).

Table 3 shows the energy content of the school *almuerzo* during the follow-up period. For both groups, <20% of the children met the recommendations related to “healthy school meals,” with > 50% of children within both groups displaying school *almuerzo* energy values higher than the one recommended (~150–200 kcal higher), during the follow-up period. The school *almuerzo* energy value among the children who only brought their own and those who brought their own in addition to money for extra food was similar.

Finally, we assessed the change in grams and milliliters in the percentage of calories in each food group comprising the school meals during the follow-up period and, to compare the groups, we used the ANOVA model for repeated measures, thus generating in the end predictive values using the marginals (Table 4). For children who brought fruits, between 20% and 30% of the calories present in their school meals came from this food group, roughly equal to one serving of fruit; in the CG, the fruit-related percentage of calories in the school meals increased after six months but decreased after 12 months (*p* = 0.017). In the IG, the amount in milliliters of plain water present in the school meals went up during the follow-up period (*p* = 0.044), with the children bringing an average of 500 mL of water. As for sweet drinks, despite the percentage of children who brought them in their school meals during the follow-up period decreasing, we noticed a considerable increase, both in the CG and in the IG, in the amount in milliliters of these drinks among children who did bring them in their school meals (*p* = 0.002 and *p* = 0.019, respectively). When it came to the energy-value percentage of these drinks in the school meals, there were changes: this percentage went up in the CG after 6 months and then dropped to the baseline measured values; however, in the IG it increased after 12 months (*p* = 0.024 and *p* = 0.047 in the CG and the IG, respectively). Among the children who brought sweet drinks, these accounted for between 26% and 33% of the school meals’ energy values during the follow-up period. In the CG, among the children who brought cookies in their school meals, these accounted for between 42% and 51% of the school meals’ energy values. However, in the IG, the energy-value percentage resulting from these cookies dropped from 40.9% to 24.2% and 27.5% after 6 and 12 months, respectively (*p* < 0.001).

## 4. Discussion

This study highlighted the impact of an intervention, both in-person and remote, on children’s eating habits during their stay at school-with an emphasis on assessing the quantity and quality of the foods that comprised the school meals—and on the availability of money to buy extra food from the school store. Our formative intervention neither succeeded in increasing the frequency with which parents provided children with school meals nor in altering the energy value or the quality of foods and beverages comprising that meal, which indicates that the children still have to deal with unhealthy foods. Improper eating habits are one of the risk factors related to schoolchildren’s becoming overweight and displaying obesity and there is a concern about the excess amount of energy school *almuerzo* may bring to the total diet of children [12,13].

There are many factors that influence food choices for a school *almuerzo*, such as the culture, socioeconomic status or child age. Our study showed that, during the baseline period, 88% of the children brought their own school *almuerzo* daily. Nevertheless, 43% of the children brought money to buy food at school. We did not notice any differences when we compared the energy value of meals among the children who only brought their own *almuerzo* to that of children who brought their own *almuerzo* in addition to money for extra food at school. Yet, this information does not back the hypothesis that the children from the latter group might consume more calories during a given day, nor does it eliminate the recommendation that children bring to school a home-cooked meal, thus removing the requirement for money to buy extra food at school. This study did not evaluate the foods that were sold in the school store; however, foods and drinks with high energy values are regularly sold, such as cookies, industrial juices or sweet drinks, while fruits, vegetables, plain water or healthy dishes are available in limited quantities [14]. In this study, it was possible to modify the percentage of kcal from cookies in the IG children, while in the CG group the percentage remained unchanged. This type of food generally has a high energy content, with a low contribution in nutrients; this type of food contributes to raising the energy content of the school meal, so its consumption should be limited. Although the selling of certain foods has been regulated in schools in Mexico, the guidelines have not been entirely followed [14]; therefore, children cannot enjoy healthy foods and drinks.

Among the factors that lead to the development of obesity is the consumption of foods and beverages that are high in sugar and calories. The application of taxes to sweetened beverages is a public health strategy proposed in the international context, as a priority component to prevent and control obesity and diet-related non-communicable diseases. The World Health Organization recommends that governments impose taxes on sweetened beverages that increase the price by at least 20% to reduce consumption and improve health [15]. In Mexico, as of 2014, it was approved to apply a tax of 1 peso per liter to soft drinks and 8% of the cost to non-essential foods with high energy density [16]; with this tax there has been a small decrease of −5.4 g/week per capita (relative reduction of −5.3%) in the purchases of these foods between the years of 2016 and 2018, compared to the years 2008, 2010 and 2012; characteristics such as urban area, households with children, the intermediate educational level of the head of the family, influenced the largest reduction in purchases (−6.9%, −7.0% and −9.9% respectively) [17]. The calories contributed by beverages and non-essential foods had a decrease of 8.5% and 5.4% respectively; total calories, saturated fat and sodium increased slightly [18]. Although the results are positive, the implementation of the tax policy in isolation will hardly modify the trends in the obesity epidemic in the different age groups; therefore, complementary actions that promote the selection, purchase and consumption of tax-free foods with high nutrient content and moderate or low caloric density should be considered.

On a similar note, the school *almuerzo* brought by the children lacked fruits, vegetables and plain water. The foods provided by the parents in the school meals were not very healthy. Fruits, vegetables and plain water must be included on a daily basis in a healthy school meal [6]. This study did not evaluate the availability of drinking water fountains in schools; however, a low frequency of water consumption with drinking fountains has been reported, due to a distrust of the origin of the water, so comprehensive actions are required to favor their consumption [19]. However, this measurement bias on water consumption from drinking fountains is not differential, since the information was collected in the same way in the intervention and control groups. The school *almuerzo* represents the food consumed by children during their stay at school and must include unlimited plain water, one or more servings of vegetables and/or fruits, along with one serving of cereal (whole), accompanied by a protein-containing type of food, be it an animal or vegetable source [6]. Based on this study, we reported that, in the meals that contained vegetables, these were only one part of the ingredients—as was the case with sandwiches or cakes—and very seldom included as independent servings, which led to vegetable consumption being scarce. Moreover, we noticed how often sweet drinks were included in the meals, in high quantities, which led to obesity, metabolic disorders, insulin resistance or diabetes mellitus, from increasingly early ages [20,21]. Although in both groups the amount of sweet drinks in the meals dropped, it remains important to place together interventions focused on altering and eliminating this habit within families.

The energy value contained in the school meals brought from home and consumed during the break was of 395 kcal (281–527). These values agree with the ones in previous studies, which speak about a consumption level of between 401 and 433 kcal, which accounts for 24% of the daily energy-value recommendations for pupils, according to age, sex and light physical activity [6,14]. We feel it is important to reiterate that these numbers only revolve around the food brought from home, without considering that a high percentage of pupils brings money to buy extra food at school. The study leaves the hypothesis that those who brought both school *almuerzo* and money on a regular basis during the study have a higher risk of obesity, a hypothesis that was not analyzed in the groups. However, a study about the frequency, amount and quality of foods consumed at eating occasions and their impact on total energy intake in the diet of Mexican children between 6 and 13 years old reported that for each extra snack meal, the amount of calories increases between 191 and 289 kcals in the daily energy intake [5].

In this study, a low percentage (7%–20%) of school *almuerzo* meals displayed an energy value within the limits of the recommended one (250–340 kcal per day) throughout the follow-up period, with > 50% of the children in the two groups, both at the beginning and after 6 and 12 months, having brought to school a meal whose energy value exceeded the one recommended (>340 kcal per day) [6]. This reflects an energy value excess of ~150–200 kcal in the school meals prepared by parents. If this excess of energy value is constantly consumed, children could risk becoming overweight and displaying obesity. According to our data and to the data from other studies, this bad habit could be attributed to the parents’ lacking information or time to plan, buy and prepare meals [6,14], likewise, to the influence of the mass media that influence the consumption of sweet drinks and industrialized foods, which are found on the shelves of stores that sell food. However, another aspect that requires more research is if this eating occasion must be assessed as one main meal or as a snack meal; this eating occasion is similar to the “*almuerzo*” that is one common meal in the adult Mexican population, that includes foods such as “*tacos*”, meat or sandwich and has a greater contribution in nutrients and energy than a snack meal. The time between breakfast and lunch is approximately seven hours in the schedule of meal occasions in Mexico. If the school meal is considered as one main meal, the dietary recommendations for this eating occasion should be reconsidered, allowing its contribution to the daily energy and nutrients to be greater.

Our intervention did not succeed in altering the frequency with which the parents provided children with school *almuerzo*. However, it did manage to maintain the percentage of children who only brought their own school *almuerzo*, a thing that could be attributed to the families’ current reluctance to alter their eating habits, seeing as it involves their reorganizing their nourishment process, starting with the planning, buying and preparing of meals for every day of the week. On a different note, we noticed that giving money to children has become a habit among parents. During the follow-up period, in both groups, >40% of the children carried money, of which ~10% of the parents had only opted to provide them with money, but not with a school meal. Altering habits is a complex operation that requires long-term work on behalf of both the parents and the children, teachers and principals [4].

Both in the IG and CG, the percentage of children who brought sweet drinks in their meals decreased. Nevertheless, during the follow-up period, the children who did bring these drinks augmented their quantities in milliliters, thus enabling the energy value of the meals to augment (26%–33%) and potentially lead to the children’s becoming overweight and displaying obesity. We wish to highlight that, in the IG, the percentage of children who brought plain water did not change; however, during the follow-up period, this group of children increased the quantity in milliliters, probably because this was a habit that could be altered easily, seeing as it did not involve much time, preparation or higher costs. However, the increase in milliliters of sweet drinks or plain water could be related to the season and to the rise in temperature [22].

Throughout the follow-up period, <44% of the children brought one serving of fruits, <25% brought one serving of vegetables and <53% brought plain water in their school meals. These habits should be changed from the homes. All children should bring fruits, vegetables, and one serving of proteins, cereal and plain water daily in their meals [6,23]. In Mexico, the recommendation of the distribution of the total energy intake for the child population is that they have three meal times (breakfast, lunch and dinner, 25%, 30% and 15%, respectively) and two snacks, with a contribution 15% ± 5% each, (a snack during the stay at school) [24]; however, it is possible that the food intake throughout the day is being redistributed, the children leave home early so they do not have breakfast according to the recommendation, so the children could be consuming a higher percentage of energy during school *almuerzo*; further research is required on the subject. In Mexico, studies report that schoolchildren enjoy at least five moments during their school day in which they can eat, and that children have bad eating habits [25,26]; hence, the importance of devising efficient educational strategies to change feed quality.

The present study failed to improve the quantity and quality of food that children bring from home; therefore, future interventions should focus on enhancing the quantity and quality of *almuerzo* meals brought to school and devise comprehensible guides for the proper quantities and servings as per children’s age, sex and physical activity to improve their eating habits. During the next interventions, it is important that parents’ role in selecting, buying, preparing and serving children’s meals be strengthened, both inside and outside homes, with the purpose of acquiring healthy eating habits. In the same manner, children must become involved in these interventions, learn to select healthy foods and aid in preparing them. As part of this intervention, we suggest that community interventions related to changes in eating habits be more concentrated and last longer to achieve significant and permanent changes. It is within the family that children learn and adopt eating conducts, the parents playing the part of health promoters and role models, thus influencing the children’s future food choices [4].

Among the limitations of our study, there was the low rate of agreement (50%) to participate in the study for both groups not having investigated the reasons behind the parent’s reluctance to participate. On a similar note, we noticed a low participation of parents in the website-surfing activity and in the in-person workshops. Also, attending the in-person workshops required the parents’ taking time to go and attend these sessions at school, in a very complex city mobility wise. After all, a high percentage of mothers and fathers were working. The intervention included in this study and characterized using various means of conveying messages and information to parents and their children was aimed at parents seeing as they are the ones responsible for shaping their children’s eating habits. Internationally, there is evidence of the potential that multilevel interventions have in enhancing the contents of school meals by actions targeting parents, children and teachers, and aiming to improve the quantity and quality of foods consumed by pupils [27,28,29]. Some researchers believe that the programs meant to enhance the quality of school meals at a pre-school and school level ought to focus on increasing the consumption of fruits and vegetables, in addition to promoting the inclusion of various food groups [28,30,31]. Another limitation of our study is that we did not ask the children about how much of the school *almuerzo* they consumed, whether they gave it away or returned it home. Likewise, we did not measure whether or not children bought food at school, nor the type of food they bought. We also did not assess the daily intake of energy and nutrients; although this was not the objective of the study, it could help to know if children are consuming extra calories and to quantify the excess.

## 5. Conclusions

The quantity and quality of the foods and beverages that comprise pupils’ school *almuerzo* are not very healthy because of their high energy values, low contents of fruits, vegetables, and plain water, and high contents of sweet drinks. Our intervention did not succeed in modifying the frequency of home-cooked meals; however, it had a positive impact on the consumption of plain water. The results obtained in this study indicate the need to research and implement targeted intervention programs and nutrition education to help change children’s food choices as a strategy for preventing overweight and obesity among children.

## Figures and Tables

**Table 1:** Baseline characteristics of the study population.

Characteristics	Intervention	Control	p ^‡^
	*n* = 225	*n* = 177	
	Mean ± SD	Mean ± SD	
**Age (y)**	7.9 ± 1.2	8.1 ± 1.2	0.152
**Sex (Female), n (%)**	101 (44.9)	89 (50.3)	0.282
**Anthropometric measures**			
Weight (kg) ^†^	29.3 ± 5.0	30.0 ± 5.1	0.154
Height (cm) ^†^	126.3 ± 7.3	127.3 ± 7.4	0.171
BMI z-score ^§^	0.85 ± 1.4	0.99 ± 1.3	0.290
**Classification of BMI ^§^, n (%)**			
Normal (−2 < z-score < 1)	129 (57.3)	87 (49.2)	
Overweight (1 ≤ z-score < 2)	52 (23.1)	45 (25.4)	
Obesity (z-score ≥ 2)	44 (19.6)	45 (25.4)	0.224
**Brought school *almuerzo*, n (%)**	203 (90.2)	151 (85.3)	0.132
**Brought money to buy food at school**	96 (42.7)	76 (43.7)	0.840
**Education level of the mother, n (%)**			
Secondary or less	42 (20.9)	24 (14.1)	
High school or technical school	76 (37.8)	77 (45.3)	
College career or postgraduate	84 (41.3)	69 (40.6)	0.162
**Socioeconomic status (*n* = 356) n (%)**			
Lower	63 (32.3)	42 (26.1)	
Medium	76 (39.0)	53 (32.9)	
Higher	56 (28.7)	66 (40.1)	0.052

^†^ Means adjusted by age and sex. ^§^ World Health Organization 2007. ^‡^ Student’s *t*-test and Pearson’s Chi-squared test. BMI, Body mass index.

**Table 2:** Energy content and macronutrients from the school *almuerzo*, at the beginning of the study.

Macronutrients	All *n* = 354 Median (25*p*, 75*p*)	Intervention *n* = 203 Median (25*p*, 75*p*)	Control *n* = 151 Median (25*p*, 75*p*)
Energy (kcal)	395 (281, 527)	396 (269, 523)	394 (289, 538)
Carbohydrates (g)	57 (35, 79)	55 (33, 76)	60 (36, 83)
Carbohydrates (%)	57 (46, 70)	56 (44, 70)	58 (49, 70)
Proteins (g)	12 (8, 17)	12 (7, 17)	12 (8, 17)
Proteins (%)	12 (9, 15)	11 (9, 16)	12 (8, 14)
Fat (g)	13 (7, 20)	13 (7, 20)	13 (6, 20)
Fat (%)	29 (18, 41)	29 (18, 42)	29 (17, 39)
Fiber (g)	2.9 (1.1, 5.0)	2.7 (1.1, 4.7)	3.0 (1.1, 5.6)

Mann–Whitney U test, value *p* > 0.05; *p*, percentile.

**Table 3:** School *almuerzo* energy value during the follow-up period.

	Intervention (*n* = 193)				Control (*n* = 150)			
	Baseline n (%)	6 Months n (%)	12 Months n (%)	*p* ^‡^	Baseline n (%)	6 Months n (%)	12 Months n (%)	*p* ^‡^
**Categories According to Energy Value**								
No school *almuerzo*	21 (10.9)	16 (8.3)	32 (16.6)		23 (15.3)	17 (11.3)	31 (20.7)	
Low-calorie (<250 kcal)	34 (17.6)	31 (16.1)	29 (15.0)		20 (13.3)	30 (20.0)	25 (16.7)	
Healthy (250 to 340 kcal) ^¥^	32 (16.6)	38 (19.7)	28 (14.5)		24 (16.0)	24 (16.0)	11 (7.3)	
High-calorie (>340 kcal)	106 (54.9)	108 (56.0)	104 (53.9)	0.012	83 (55.3)	79 (52.7)	83 (55.3)	0.030
**Energy value (kcal)**	Median (25*p*, 75*p*)	Median (25*p*, 75*p*)	Median (25*p*, 75*p*)		Median (25*p*, 75*p*)	Median (25*p*, 75*p*)	Median (25*p*, 75*p*)	
<250 kcal	171 (98, 224)	176 (95, 217)	137 (100, 186)		194 (111, 231)	152 (116, 216)	183 (125, 229)	
250 to 340 kcal ^¥^	304 (272, 323)	306 (284, 325)	302 (273, 324)		296 (276, 319)	294 (261, 315)	295 (284, 315)	
≥340 kcal	480 (421, 582)	473 (395, 562)	477 (410, 595)		508 (399, 626)	494 (423, 595)	486 (403, 601)	
**Children who only brought their own meal (kcal)**	426 (283, 542)	394 (289, 500)	401 (310, 547)		415 (316, 547)	391 (262, 498)	420 (351, 551)	
**Children who brought their own meal + money (kcal)**	380 (269, 482)	349 (292, 462)	411 (267, 527)		374 (290, 513)	432 (142, 573)	402 (236, 562)	

^¥^ National Institute for Public Health. Technical Document of Recommendations for Nourishment Guides in Public Primary Schools. Characterization of the school environment among primary schools in states of the Mexican Republic and Recommendations for a “Healthy school meal,” 2010. ^‡^ Cochran’s Q test.

**Table 4:** Change in grams and milliliters, and energy-value percentage in the food groups comprising the school *almuerzo*, during the follow-up period.

	Intervention				Control			
	Baseline Mean (95% CI) *n* = 203	6 Months Mean (95% CI) *n* = 195	12 Months Mean (95% CI) *n* = 162	*p* *	Baseline Mean (95% CI) *n* = 151	6 Months Mean (95% CI) *n* = 144	12 Months Mean (95% CI) *n* = 119	*p* *
**Fruits, n (%)**	82 (40.4)	80 (41.0)	71 (43.8)	0.787	50 (33.1)	62 (43.1)	53 (44.5)	0.102
Grams	143 (119 to 167)	180 (156 to 204)	152 (125 to 178)	0.112	146 (123 to 167)	174 (156 to 193)	140 (120 to 160)	0.051
% of kcal	30.9 (25.7 to 36.1)	24.6 (19.4 to 29.7)	26.7 (21.0 to 32.4)	0.269	21.0 (14.3 to 27.6)	31.8 (26.1 to 37.6)	19.8 (13.7 to 25.9)	0.017
**Vegetables, n (%)**	34 (16.8)	44 (22.6)	39 (24.1)	0.180	27 (17.9)	36 (25.0)	27 (22.7)	0.319
Grams	91 (38 to 145)	116 (74 to 156)	103 (53 to 152)	0.788	90 (33 to 146)	118 (72 to 164)	77 (16 to 138)	0.572
% of kcal	9.7 (0 to 22.4)	11.4 (1.8 to 21.1)	18.9 (7.3 to 30.5)	0.587	3.2 (0 to 12.7)	25.8 (0 to 13.7)	19.5 (9.2 to 29.8)	0.106
**Water, n (%)**	98 (48.3)	102 (52.3)	86 (53.1)	0.601	47 (31.1)	63 (43.8)	45 (37.8)	0.081
Milliliters	454 (407 to 501)	510 (464 to 555)	546 (496 to 595)	0.044	445 (366 to 525)	547 (483 to 610)	556 (477 to 634)	0.132
**Sweet drinks, n (%)**	83 (40.9)	77 (39.5)	48 (29.6)	0.061	89 (58.9)	66 (45.8)	41 (34.5)	<0.001
Milliliters	384 (334 to 433)	399 (349 to 450)	509 (441 to 576)	0.019	407 (363 to 451)	467 (415 to 518)	571 (501 to 641)	0.002
% of kcal	26.3 (23.5 to 29.1)	27.7 (24.9 to 30.1)	32.7 (28.8 to 36.5)	0.047	27.7 (24.0 to 31.6)	32.4 (27.9 to 36.9)	28.1 (32.1 to 44.1)	0.024
**Dairy products, n (%)**	117 (56.7)	114 (58.0)	90 (55.6)	0.901	91 (60.3)	72 (50.0)	64 (53.8)	0.201
Grams	79 (63 to 95)	77 (61 to 93)	92 (74 to 109)	0.450	60 (42 to 77)	85 (65 to 104)	66 (45 to 86)	0.198
% of kcal	27.1 (23.7 to 30.6)	26.4 (22.9 to 29.7)	30.1 (26.2 to 33.9)	0.349	21.7 (17.3 to 26.1)	24.0 (19.0 to 29.0)	25.1 (19.8 to 30.4	0.642
**Cookies, n (%)**	44 (21.7)	32 (16.4)	33 (20.4)	0.391	26 (17.2)	23 (16.0)	20 (16.8)	0.958
Grams	55 (43 to 67)	35 (21 to 48)	38 (24 to 51)	0.090	58 (42 to 75)	63 (44 to 81)	51 (32 to 68)	0.662
% of kcal	40.9 (28.8 to 70.3)	24.2 (12.8 to 39.7)	27.5 (16.3 to 38.7)	<0.001	42.2 (26.4 to 52.7)	48.3 (22.2 to 73.2)	51.2 (22.5 to 71.2)	0.664

* ANOVA model of repeated measures and predictive marginals.

## Data Availability

The data presented in this study are available on request from the corresponding author. The data are not publicly available.

## Supplementary Materials

The following are available online at https://www.mdpi.com/2075-1729/11/3/253/s1, Table S1: Work topics on the website and phone messages aimed at parents.
