# Supplementary material for: Impact of a Nutrition-Related Community Intervention on the Quantity and Quality of Children’s School almuerzo
Source: Life (Basel). 2021 Mar 19;11(3):253. doi: 10.3390/life11030253 (PMC8003566; doi:10.3390/life11030253)
Supplement: Supplementary file 1 [file life-11-00253-s001.pdf]

life

MDPI

Article

Impact of a Nutrition-Related Community Intervention on the Quantity and Quality of Children's School *almuerzo*

Jenny Vilchis-Gil [1,2,*], Miguel Klünder-Klünder [3,4], Ximena Duque-López [5], Gloria Martínez-Andrade [6], Andrea Martínez-Almaraz [1], Brenda Beristain-Lujano [1] and Samuel Flores-Huerta [1]

[1] Epidemiological Research Unit in Endocrinology and Nutrition, Hospital Infantil de México Federico Gomez, Ministry of Health (SSA), 06720 Mexico City, Mexico; andy_270293@hotmail.com (A.M.-A.); brendaberistain10@gmail.com (B.B.-L.); sflores@himfg.edu.mx (S.F.-H.)

[2] Medicine Faculty, National Autonomous University of Mexico, 06320 Mexico City, Mexico

[3] Deputy Director of Research, Mexico Children's Hospital Federico Gómez, 06720 Mexico City, Mexico; miguel.klunder@himfg.edu.mx

[4] Research Committee, Latin American Society for Pediatric Gastroenterology, Hepatology and Nutrition (LASPGHAN), Mexico City, Mexico

[5] Infectious Diseases Research Unit, Pediatric Hospital, Mexican Institute of Social Security, 06600 Mexico City, Mexico; ximena.duque@imss.gob.mx

[6] Epidemiological and Health Services Research Unit, Mexican Institute of Social Security, 06600 Mexico City, México; gloria.martineza@imss.gob.mx

* Correspondence: jvilchis@himfg.edu.mx, Tel.: +52-55-5228-9917 (ext. 4510)

Citation: Vilchis-Gil, J.; Kl, M.; Duque-L, X.; Mart, G.; Mart, A.; Beristain-Lujano, B.; Flores-Huerta, S. Impact of a Nutrition-Related Community Intervention on the Quantity and Quality of Children's School *almuerzo*. *Life* **2021**, *10*, x. https://doi.org/10.3390/xxxxx

Received: 04 February 2021

Accepted: 14 March 2021

Published: date

Publisher's Note: MDPI stays neutral with regard to jurisdictional claims in published maps and institutional affiliations.

Copyright: © 2021 by the authors. Submitted for possible open access publication under the terms and conditions of the Creative Commons Attribution (CC BY) license (http://creativecommons.org/licenses/by/4.0/).

Abstract: Foods and beverages that schoolchildren carry in their lunchboxes have high energy values but lack plain water, fresh fruits and vegetables. A nutrition-related community intervention on the quantity and quality of school *almuerzo* was performed, in which four primary schools participated, as part of two groups: 225 children in the intervention group (IG) and 177 children in the control group (CG). The parents from the IG had access to a website where they could consult information on eating habits and physical activity or school *almuerzo* menus. They were sent weekly text messages on their mobile phones and attended in-person sessions. Anthropometric measurements and surveys were performed in both groups at the start of the study, as well as after 6 and 12 months. The school *almuerzo* was assessed by recording foods that the children brought in their lunchboxes. At baseline, 88% of children brought a school *almuerzo*, 37% fruit, 17% vegetables, 40% plain water and 50% sweet drinks. In both groups, 50% of children brought a school *almuerzo* with an energy value above the recommended value (> 340 kcal) during follow-up; however, the percentage of children who brought sweet drinks decreased ($p < 0.05$), with sweet drinks contributing between 26% and 33% of the calories in the school *almuerzo*. In the IG, the quantity in milliliters of plain water increased at the end of the follow-up period ($p = 0.044$). From the point of view of food-and-beverage quantity and quality, school *almuerzo* were unhealthy for both groups. The intervention failed to increase the frequency with which parents provided children with school *almuerzo* or enhance the quality of the latter.

Keywords: child; school *almuerzo*; early intervention; prevention; obesity

Table S1. Work topics on the website and phone messages aimed at parents

Website Topics	Examples of Suggested Activities for Parents from the Topics on the Website	Text Messages Sent to Parents by Mobile Phone
1. What is obesity? Why are there more obese people now than before?	- Limit the intake of energy-dense foods, with a large amount of carbohydrates and fat, for example sweet bread, desserts, snacks, sweets etc.<br>- Increase the consumption of fruits, vegetables, legumes and whole grains.<br>- Decrease car use and increase daily walking.	**M1.** Welcome, you and your child are participating in the project "*Aliméntate y Actívate Sanamente*" at the Hospital Infantil de México Federico Gómez. Please save this phone number, which is where the rest of the messages will come from.<br>**M2.** Our habits may show that the balance between energy consumption and expenditure has been broken, excess food consumption is stored as fatty tissue, until it becomes obesity, which is associated with the development of high blood pressure and type 2 diabetes.
2. Obesity and its relationship to chronic degenerative diseases	- Help us prevent. Help your children adopt healthy eating and physical activity habits and be mindful of what they eat.<br>- Prepare healthy dishes and reduce high-calorie temptations.	**M3.** The abundant and unhealthy food, as well as the reduction of physical activity or exercise, are factors that affect the health of children and adults.<br>**M4.** When the body accumulates more fat than expected, obesity appears; its treatment is slow, ineffective and expensive, but it can be prevented. Get informed!
3. Lifestyles, feeding, activity/sedentary behavior, and health monitoring with regular measurements	- Offer your family a variety of vegetables, fruits, and whole grains daily, serve reasonable-sized portions and drink plain water.<br>- Help your kids stay active, take them to the park, play with them.<br>- Get regular nutritional status assessments and have blood tests done once a year.	**M5.** With obesity, diseases such as type 2 diabetes, hypertension and other heart diseases appear. Before adults suffered from them, now they have reached children. Careful!<br>**M6.** Three small changes to take care of our health: 1. Choose healthy foods daily, 2. Exercise every day, 3. Measure yourself at least once a year.
4. Lifestyle and health. We stay as we are or we make an effort to improve the health of the family and of each of its members	- To buy food, make a list, start by writing down the green leafy vegetables and vegetables, in sufficient quantity to prepare salads during the week, or to steam them and accompany the main dish. Don't forget to write down the fruits. For liquid foods, look for semi or skim milk.<br>- Find a place near where you live for you and your family to get some exercise.	**M7.** "Measuring yourself on what": 1. Do not overeat or ingest junk food or sweet drinks; 2. Know if your weight is normal; 3. Get blood tests once a year.<br>**M8.** Our health and that of our children depends on what we practice daily at home. Let's make a commitment to take care of it. Let's start the change! Let's buy healthy food to prepare healthy food at home.

Website Topics	Examples of Suggested Activities for Parents from the Topics on the Website	Text Messages Sent to Parents by Mobile Phone
5. Eating breakfast and bringing school meal to school are essential for health and school performance of children	- It is important for your child to eat breakfast daily before going to school. Wake up early to have enough time for breakfast.<br>- Send your child a healthy school meal every day. Invite your child to help prepare his school meal.<br>- Avoid or reduce the money you give your child to buy food at school, as it is better for them to eat the healthy meal you send.	**M9.** Children who skip breakfast perform less in their classes. Get into the habit of eating natural foods for breakfast. Avoid foods rich in refined flours.<br>**M10.** School meals are not a substitute for breakfast. Invite your child to participate in making the meals. See the school meals options on the website: http://himfg.com.mx/interna/dirinvestiga/alas.php
6. Physical activity is much more than expending energy	- Help your children stay active. Do physical activities that you can do as a family, such as walking, biking, rollerblading, going to museums, the park, shopping, or doing any other physical activity together.<br>- Ask your children to help with the housework. Encourage responsibility and housekeeping by having your children help wash the car, clean up their bedroom, etc.<br>- Establish a regular schedule for physical activity as part of your daily activity program.	**M11.** Children are by nature active. Encourage your children to move. Play or move around with them for at least 30 minutes daily.<br>**M12.** Getting active daily with your child brings great benefits. 30 minutes of exercise daily improves metabolic disorders in sedentary people.
7. Sedentary lifestyle, a risk for health	- Do not put a television in the child's room or in an area where food is eaten.<br>- Accompany your children to ride a bike, play, jump, dance, walk, run, jump rope, since this age is when they begin to form healthy habits.<br>- To travel short distances, prefer to walk instead of using a car. If possible, use stairs instead of an elevator.	**M13**. There is a risk of sedentary lifestyle when you spend more than 2 hours a day in front of a screen and when you travel short distances by car instead of walking.<br>**M14.** Decrease sedentary lifestyle. Avoid letting the TV stay in the bedroom and kitchen. If the destination distance is walkable, it would be a good decision to walk it.
8. Measure yourself. Self-measurement is much more than knowing your weight	- Measure yourself in the amount of food that you are going to eat, always thinking about the needs regarding age and sex.<br>- Dads motivate their children to do physical activity daily (minimum 30 minutes a day). Evaluate the amount of exercise you do in the week.<br>- Regularly evaluate your nutritional condition; measuring ourselves will help us identify if we have a healthy weight or not.	**M15.** Measure the weight, height, and waist circumference of the whole family twice a year. At each meal time, think about whether you exceed consumption.<br>**M16.** During the year-end festivities, the opportunities to eat and drink increase; if you take care of your diet at this time of year, there will be no January cost… neither in your pocket nor on the scale.

Website Topics	Examples of Suggested Activities for Parents from the Topics on the Website	Text Messages Sent to Parents by Mobile Phone
9. Plain water is the healthiest	- Always keep a pitcher of plain water in the refrigerator or on the table.<br>- When they eat, offer plain water instead of any sweetened drink, or make fruit water without adding sugar.<br>- Limit the consumption of sweetened and / or industrialized beverages in the family.	**M17.** How to provide our body with the water it needs? drinking natural and plain water.<br>**M18.** Put a pitcher of plain water on your table every day. You can add fruit but not sugar. Juices, milk, and soda are not equivalent to water.
10. Not all the energy that we consume is the same in terms of health	- Buy food according to the menu plan for the week. There are no prohibited foods, but it is recommended that fresh natural foods and whole grains be preferred.<br>- Reduce the consumption of processed foods, cold cuts, ready-to-eat food. They are generally rich in sugar, fat or salt.<br>- Invite your children to help you prepare foods such as salads, which should be on your table each day.	**M19.** The body uses the energy it gets efficiently. If it lacks, it adapts to survive. If it is left over, it is saved as adipose tissue.<br>**M20.** Our body uses the calories from natural foods better than from some processed or industrialized foods, since their nutrients have been modified.
11. Carbohydrates. Health benefits and risks	- Buy whole grains like rice, oatmeal, corn *tortillas*, etc., and other foods like potatoes, beans or vegetables.<br>- Limit the purchase of foods that contain simple sugars such as sweet bread, cakes, jam, cookies, packaged cereals, soft drinks, industrialized juices, etc.<br>- Invite your children to help with the preparation of dishes, so they will feel happy about their participation.	**M21.** Natural foods like whole grains, legumes, fruits and vegetables contain complex or healthy carbohydrates. Prefer them!<br>**M22.** Simple carbohydrates are a health risk. They are found in processed foods like cookies, jams, sodas, juices, etc. Buy them less.
12. Fiber. Health benefits and risks	- Include foods rich in fiber, such as vegetables, fruits, legumes, and whole grains, on your shopping list.<br>- Consume the whole fruit and, if possible, with the peel.<br>- Consume 1-2 servings of vegetables without cooking, that is, in their fresh and raw form.	**M23.** Eating foods rich in fiber will help improve your digestion, slow the absorption of nutrients, and prolong your satiety. Consume them.<br>**M24.** Fiber is mainly found in foods like fruits, vegetables, and legumes like beans, lentils, and lima beans. Consume them daily.
13. Importance of eating fruits and vegetables	- At home, place the fruit where you can see it, so there will be a better chance of eating it.<br>- Every day and at every mealtime, put vegetables or fruit.<br>- Fruit looks good for breakfast, snacks or dinner. Vegetables can be half the food on the plate. Do not forget that they are preferably	**M25.** Include fresh fruits and vegetables on your shopping list. Consume them at every mealtime. A few more vegetables each time and in each dish are better.<br>**M26.** Salads are the best way to eat fruits and vegetables. Make up your own salads combine various types of green leaves, tomato,

Website Topics	Examples of Suggested Activities for Parents from the Topics on the Website	Text Messages Sent to Parents by Mobile Phone
	fresh.	cucumber, fruit, and add nuts or cheese.
14. Lipids. Health benefits and risks	- Use simple cooking methods that do not require a lot of oil: steamed, grilled, roasted, oven, in its juice.<br>- Avoid putting creamy toppings, mayonnaise, or cream on the table.<br>- Remove excess fat from food; draining or absorbing it with a napkin.<br>- Eat fresh fish at least 2 times a week.	**M27.** Reduce the consumption of fried, battered or breaded foods. Increase steaming, roasting and baking preparations. Avoid foods that say hydrogenated fat on the label.<br>**M28.** Fish, nuts and avocado contain beneficial fat for the body. Meat, sweet bread, butter contain fats that are risky for health. Drink low-fat milk.
15. Proteins in food. Health benefits and risks	- Include the purchase of skim milk, fish, chicken without skin, fresh cheeses.<br>- Consume 1 serving a day of legumes (1/2 cup of beans, lentils, lima beans, chickpeas, soybeans, beans, etc.). These are a good source of protein.<br>- Eat fish twice a week. There are inexpensive species such as saw, dogfish, band, mojarra, sole, surimi, etc. Prefer it over red meat.<br>- Only include cold cuts once a week, no more. If you can avoid them, the better.	**M29.** Offer one or two servings of protein foods at each mealtime daily. Combine those of animal and vegetable origin.<br>**M30.** Include in your child's school meal a serving of: a) Proteins such as cheese, meat, fish or beans, b) Cereal such as bread or tortilla and c). Don't forget to include fruits, vegetables, and plain water
16. Table salt in food. Health benefits and risks	- Remove the salt shaker from the table; many times we add salt only through inertia.<br>- If you cook at home, you can control the ingredients of your meals. Preparing your own food allows you to limit the amount of salt. Prefer the use of spices, herbs, garlic, lemon juice and seasonings for food preparation.<br>- Look for foods with labels that say "low sodium," "reduced sodium," or "no added salt."	**M31.** To reduce the salt we eat, just add the necessary salt when cooking. Use spices, herbs, garlic or pepper to season. Avoid putting the salt shaker on the table.<br>**M32.** Sausages such as bacon, chorizo, ham and canned foods are processed foods with high sodium content; reduce your consumption. Choose fresh food.
17. Vitamins and minerals. Health benefits and risks	- Ensure that each meal time has a food from the recommended groups.<br>- Learn the importance of eating a variety of foods to ensure that you are getting all the necessary nutrients.	**M33.** Vitamins and minerals are essential for health and are not manufactured by the body. We need include in our diet all food groups.<br>**M34.** Buy enough vegetables, fruits, whole grains, legumes, and animal products to get enough vitamins and minerals. Vary the foods.

**Website Topics**	**Examples of Suggested Activities for Parents from the Topics on the Website**	**Text Messages Sent to Parents by Mobile Phone**
18. Family behavior during food consumption at home	- Eat with your children when possible.<br>- Ask the children to help set the table by asking them to put the best healthy drink.<br>- Turn off the television and cell phones, chew food slowly and thoroughly, get up from the table when you finish eating.	**M35.** Plain water should not be missing on your table and a daily vegetable salad should not be missing on your plate. Do not invite soda or salt to the banquet.<br>**M36.** Abundance of food on the plate invites you to eat more. Eat slowly and savor the food. During the meal avoid watching TV.
19. Planning food purchases. Learning to read labels on processed and industrialized foods	- Before buying food, plan your week's menu well and make your shopping list of foods, preferring the fresh and natural over the industrialized or packaged.<br>- Eliminate bottled soft drinks, industrialized juices, sweets, and snacks from your shopping list.<br>- When shopping for processed foods, identify that these foods are low in sugar, fat, and salt.	**M37.** Buy food according to the menus that you are going to prepare during the week. Do not forget that fruits and vegetables are required daily and at each meal time.<br>**M38.** Always read food labels to find out what they contain. Avoid buying those that have refined sugars and high fat content.
20. Integration. Create a healthy eating environment with your family		**M39.** Remember that family is the most influential factor when it comes to changing habits. Involve everyone in the choice and preparation of the family menu.<br>**M40**. We appreciate your participation and that of your child, continue with the change of eating habits and physical activity, to maintain or improve your health.
